# Retraction Note: Wogonin reversed resistant human myelogenous leukemia cells via inhibiting Nrf2 signaling by Stat3/NF-κB inactivation

**DOI:** 10.1038/s41598-022-13462-0

**Published:** 2022-05-30

**Authors:** Xuefen Xu, Xiaobo Zhang, Yi Zhang, Lin Yang, Yicheng Liu, Shaoliang Huang, Lu Lu, Lingyi Kong, Zhiyu Li, Qinglong Guo, Li Zhao

**Affiliations:** 1 State Key Laboratory of Natural Medicines, Jiangsu Key Laboratory of Carcinogenesis and Intervention, China Pharmaceutical University, 24 Tongjiaxiang, Nanjing, 210009 People’s Republic of China; 2 State Key Laboratory of Natural Medicines, Department of Natural Medicinal Chemistry, China Pharmaceutical University, 24 Tongjiaxiang, Nanjing, 210009 People’s Republic of China

Retraction of: *Scientific Reports* 10.1038/srep39950, published online 2 February 2017

The Editors have retracted this Article. After publication, concerns were raised regarding partial image duplication in Figs. 1g, 2e, 5a,b, 6b and 8e. The Authors issued a Correction^1^ to address these issues. However, additional concerns have been raised:

- p-IkBα lanes 2 and 3 in Fig. 1f. appear highly similar to p65 lanes 1 and 2 in Fig. 3d;
- IkBα lanes 4 and 5 in Fig. 1f. appear highly similar;
- IKKα lanes 1 and 2 in Fig. 1g appear to be highly similar to lanes 3 and 4;
- Fig. 6a appears to contain duplicated data patterns in the flow cytometry plots representing the 10 and 20 μM groups (between Q2 and Q3 within the groups, and in all quadrants between the two groups).

Given these additional concerns, the Editors no longer have confidence in the presented data.

Qinglong Guo does not agree to this retraction. Xuefen Xu, Xiaobo Zhang, Yi Zhang, Lin Yang, Yicheng Liu, Shaoliang Huang, Lu Lu, Lingyi Kong, Zhiyu Li, and Li Zhao have not responded to any correspondence from the Editors or publisher about this retraction.
